# Capturing socially motivated linguistic change: how the use of gender-fair language affects support for social initiatives in Austria and Poland

**DOI:** 10.3389/fpsyg.2015.01617

**Published:** 2015-10-31

**Authors:** Magdalena M. Formanowicz, Aleksandra Cisłak, Lisa K. Horvath, Sabine Sczesny

**Affiliations:** ^1^Department of Psychology, University of BernBern, Switzerland; ^2^Faculty of Psychology, Department of Social Psychology, University of Social Sciences and HumanitiesWarsaw, Poland; ^3^TUM School of Management, Technische Universität MünchenMunich, Germany

**Keywords:** grammatical gender, gender-fair language, political correctness, social change, feminism

## Abstract

Gender-fair language consists of the symmetric linguistic treatment of women and men instead of using masculine forms as generics. In this study, we examine how the use of gender-fair language affects readers' support for social initiatives in Poland and Austria. While gender-fair language is relatively novel in Poland, it is well established in Austria. This difference may lead to different perceptions of gender-fair usage in these speech communities. Two studies conducted in Poland investigate whether the evaluation of social initiatives (Study 1: quotas for women on election lists; Study 2: support for women students or students from countries troubled by war) is affected by how female proponents (lawyers, psychologists, sociologists, and academics) are referred to, with masculine forms (traditional) or with feminine forms (modern, gender-fair). Study 3 replicates Study 2 in Austria. Our results indicate that in Poland, gender-fair language has negative connotations and therefore, detrimental effects particularly when used in gender-related contexts. Conversely, in Austria, where gender-fair language has been implemented and used for some time, there are no such negative effects. This pattern of results may inform the discussion about formal policies regulating the use of gender-fair language.

## Introduction

The line it is drawn

The curse it is cast

The slow one now

Will later be fast

As the present now

Will later be past

The order is

Rapidly fadin'

…….

*For the times they are a-changin'*.

Bob Dylan, 1963

These lyrics by Dylan capture a rarely examined phenomenon in social psychology, that is, social reality changes over time and may do so even within a fairly short period. Here, we will look at socially motivated changes in language such as language reforms introduced to instigate and promote changes in social reality. To our knowledge, such language policies' effectiveness has never been examined. Such an examination would require a longitudinal approach with measurements being taken over several points of time. The disadvantage of such an approach is that the time within which changes are to happen is unspecified, which constitutes a serious challenge to the budgetary and time framework of any research. We tried to overcome this disadvantage by using cross-sectional research that compares different speech communities at different stages of implementing a specific language reform.

In our research, we focused on gender-fair language (or “non-sexist language,” UNESCO, 1999 or “gender-exclusive language,” Stout and Dasgupta, 2011), which is a type of language use that aims to represent women and men symmetrically and equally. In languages where gender-fair language was or is still a matter of debate (English in the 1990s: McConnell and Fazio, 1996; Polish and Italian in the first two decades of the twenty-first century: Mucchi-Faina, 2005; Merkel et al., 2012; Formanowicz et al., 2013), the use of feminine job titles for individual women was found to reduce women's professional credibility and evaluation, suggesting that gender-fair language and other regulations for political correctness may be counter productive. However, this conclusion may be premature as we still do not know the popular reaction to politically correct language after it has been implemented for a longer time. Positive effects of gender-fair language have been reported only for Germany, where this usage has been in practice for a longer time (Vervecken and Hannover, 2012). Hence, over time, politically correct language can be reasonably assumed to become a linguistic standard and thus may then trigger positive evaluation among its users.

We tested this assumption by comparing two speech communities where grammatical gender languages are spoken (Polish in Poland and German in Austria), which substantially differ with respect to gender-fair usage. While pertinent language reforms have been implemented and acknowledged in Austrian German, gender-fair language is rarely accepted and is often rejected in Polish. Using the same research paradigm to examine these two countries and languages representing different stages of linguistic reform, allowed the indirect study of the longitudinal effects of socially motivated language reform.

### Gender-fair language in poland and austria

In languages with grammatical gender (such as German and Polish), most human nouns and pronouns are differentiated as feminine or masculine. Therefore, the principle strategy employed to make a language gender fair is to have feminine forms of human nouns used more frequently and systematically to make female referents visible. This means masculine generics, that is, grammatically masculine forms meant to represent both genders (e.g., German *Leser*, Polish *czytelnicy* “readers, masc.”) are replaced by feminine–masculine word pairs (e.g., German *Leserinnen und Leser;* Polish *czytelnicy i czytelniczki* “*readers, fem*. *and readers, masc*.”). Additionally, feminine role names or job titles are introduced to designate female job holders explicitly (e.g., German *Psychologin* or Polish *psycholożka* “female psychologist”).

However, across the two countries and languages, differences persist in the adoption of gender-fair language. Two main reasons account for these differences. The first concerns the time of implementation. The debate about gender-fair language was a hot topic in Germany in the 1970s (Trömel-Plötz, 1978). Since then, official regulations have been adopted in German-speaking countries. The implementation of gender-fair language has progressed so far that there is even a special Microsoft add-in for gender-fair German^1^. In Austria, almost all universities and government institutions have their own guidelines for gender-fair language (e.g., University of Salzburg: Gendup, 2012; Technical University of Vienna: Arbeitskreis für Gleichbehandlungsfragen der TU Wien, 2010; e.g., Ministry of Education, Science and Culture: Bundesministerium für Bildung, Wissenschaft und Kultur, 2001; Ministry of Science and Research: Bundesministerium für Wissenschaft und Forschung, 2011). Presently, job advertisements must be phrased in a gender-fair way, e.g., with word pairs (e.g., German *Psychologin oder Psychologe* “psychologist, fem. or psychologist, masc.”) to signal that applications from both genders are welcome (Europäisches Parlament, 2009). Furthermore, according to legal regulations for equal treatment in Austria (Gleichbehandlungsgesetz, 2004; Bundesministerium für Frauen und Öffentlichen Dienst, 2009), organizations are fined if they do not advertise jobs in a gender-fair way. However, in Poland, official regulations or guidelines for gender-fair language are absent and its use is rare. According to numerous researchers, the implementation of gender-fair language has reached different stages in Austria and Poland. In an analysis of job advertisements from four European countries, only 9% of Austrian job advertisements (but as many as 83% of Polish advertisements) were found to contain masculine generics (Hodel et al., 2013).

The second reason why countries/languages differ in their implementation of gender-fair language is the relative ease with which feminine forms can be introduced. While creating feminine human nouns is fairly easy in German (mostly by adding the feminine suffix -*in* to the masculine form, e.g., *Lehrer-in* “teacher, fem.”), this is much more complicated in Slavic languages (Koniuszaniec and Błaszkowska, 2003). In Polish, feminine forms of some role nouns can easily be derived with the suffix -*ka* (e.g., *psycholoż-ka* “*psychologist, fem*.”); however, other feminine job titles with this suffix coincide with diminutive forms (e.g., Polish *fizyczka* “*physicist, fem*.” or “*little physics*”). Moreover, some feminine forms of job titles denote not only a feminine job holder but also an object (e.g., Polish *drukar-ka* “*printer, fem*.” = “*female printer*” and “*printing machine*” from *drukarz* “*printer, masc*.”). Other job titles show a semantic asymmetry: Polish *professor-ka* “*professor, fem*.” usually refers to a high school teacher, whereas the masculine form *professor* designates a prestigious academic position. Certain feminine forms also traditionally mean “wife of” rather than “female job holder” (e.g., *krawcowa* “*tailor, fem*.” or “*wife of a tailor*”).

Considering these differences, we hypothesized that reactions to gender-fair language would differ in Poland and Austria. In line with earlier findings, we assumed that reactions to gender-fair language would be more negative than reactions to traditional masculine forms in Poland, where gender-fair usage is still novel. However, in Austria, where gender-fair language is well known and fairly established, we expected gender-fair forms to trigger highly positive reactions than the traditional use of the masculine. We conducted three studies (Studies 1 and 2 in Poland and Study 3 in Austria) with a similar design to examine how the use of gender-fair language or masculine forms affected respondents' support for social initiatives (Studies 1–3) addressing gender-related (Studies 1–3), or non-gender-related topics (Studies 2 and 3).

## Study 1

### Methods

#### Participants

Study 1 was conducted in Poland via Internet. The website hosting the study was accessed by 331 individuals, 122 of whom left the first page without completing it. Thus, the final sample consisted of 209 individuals (120 women, 89 men, M age = 33.73, *SD* = 10.33 years). Of the total participants, 63% had higher education degrees, 36% secondary education, and 1% primary education.

All of the described research was conducted according to the recommendations for online research of Eynon et al. (2008). Participants were anonymous, expressed their consent to participate in the study, and were provided with the opportunity to obtain additional information on the study. The first study was a pilot study, and at the time, no institutional approval was needed in Poland for pilot studies. As the study yielded interesting results, we decided to include them in the manuscript and applied for ethical approval for subsequent studies. The study protocol was reviewed and authorized by the University of Social Sciences and Humanities Institutional Review Board (Decision record: 30/2013).

#### Measures and procedure

The study was conducted shortly before the elections of regional authorities in Poland and immediately before the deadline for the parties to submit lists of candidates to the Election Committee (in October 2010). The elections were preceded by a nationwide debate about introducing quotas for women for the election lists. Leading women in Polish society demanded a legal act according to which 50% of positions on the list would be reserved for women. This was supported with over 150,000 signatures from Polish citizens. At the time of the study, no quota system had been legally introduced; however, the topic was very popular. In fact, a legal act reserving a quota of 35% of all positions on the election lists for women and/or men was adopted shortly, thereafter by the Polish Parliament on January 5, 2011. On the website, the study was announced as a 3-min survey concerning democracy. The introduction read as follows:

“The regional elections are forthcoming, and shortly the deadline for submitting the list of candidates to the Election Committee will be reached. The legal act for a quota of 50% women candidates on the election list is under inspection by the Parliament but has not yet been decided upon. Nevertheless, women leaders (among them lawyers, psychologists, sociologists, and academics) are proposing to assign 50% of the positions on the election list to women as a societal grassroots initiative. According to this initiative, including women in the election lists would signal genuine support for gender equality in a modern Poland.”

The introduction contained the following manipulation. Half of the participants received the description of women proponents in the masculine form (Polish *adwokatów, psychologów, socjologów i nauczycieli akademickich* “lawyers, masc., psychologists, masc., sociologists, masc., and university professors, masc.”), the other half in the feminine form (*adwokatek, psycholożek, socjolożek i nauczycielek akademickich*). The original version of this manipulation (as well as of Study 2 and 3) is presented in the Supplementary Material available online. As women and men sometimes react differently to linguistic forms (e.g., Braun et al., 2005), participant gender was included as another factor.

After reading the introduction, participants answered two questions: “What are your feelings about the introduction of a gender quota in Poland?” and “What are your feelings about the social initiative presented?” They were asked to use a slider to answer these questions. The slider was preset to the mid-point position and the answers were recorded at 1-point intervals ranging from 0 (very negative) to 100 (very positive). Both items were averaged and formed a reliable scale (Cronbach's α = 0.87). This scale served as a dependent measure indicating the evaluation of the gender equality initiative^2^. To assess participants' actual support for the quota, they were also asked whether they had signed the support sheet for the quota act during the previous months. The matrix of correlation coefficients of the main variables of interest for all three Studies is available in Table 1. Finally, the participants who provided demographical data were asked for comments and were provided with debriefing information about the study.

**Table 1:** **Matrix of correlation coefficients across all three studies**.

	**Study 1**			**Study 2**				**Study 3**			
	**PG**	**PV**	**E**	**PG**	**PV**	**GI**	**E**	**PG**	**PV**	**GI**	**E**
Linguistic form	−0.03	0.04	−0.04	−0.04	0.06	0.04	−0.04	−0.02	0.01	0.01	0.14^*^
Participant Gender (PG)		0.06	0.26^***^		−0.26^***^	0.07	0.17^***^		−0.26^***^	−0.03	0.17^*^
Political Views (PV)			0.36^***^			0.00	−0.28^***^			−0.07	−0.25^***^
Goal of the Initiative (GI)							−0.17^***^				−0.10

* *p < 0.05*,

*** *p < 0.001*.

*E—in the correlation matrix refers to the Evaluation of the Initiative that is the main Dependent Variable used across the three studies*.

### Results and discussion

To test our assumptions, we conducted a regression analysis with evaluation of the social initiative as a dependent variable. In the first step, we used linguistic form (coded 0 for masculine and 1 for feminine) and participant gender (0 for male and 1 for female) as predictors, and support for the quota by signing the support sheet (0 for no and 1 for yes) as a covariate variable in the analysis^3^. The reason to use political attitudes as a covariate in our analysis was that political views can have an impact on the main dependent variable used in our studies, that is support for social equality initiatives. This assumption stems from the fact that liberals do support social equality much more than the conservatives (Jost et al., 2003). In the second step, we added an interaction term (linguistic form and participant gender), since the effects of gender-fair language may be affected by this factor (e.g., Braun et al., 2005). The results indicated that the effects of linguistic form were moderated by participant gender. An examination of the conditional effects of the linguistic form using the Hayes (2012) macro revealed that the effect occurred only among the male participants: *b* = −13.08, *SE* = 5.77; *p* = 0.02; it did not occur among the women participants: *b* = 4.70, *SE* = 4.99; *p* = 0.35. In other words, while women's evaluations of the gender equality initiative were independent of the linguistic form employed, men's evaluations were less favorable when the proponents were referred to in the feminine than in the masculine. The means and SD for all the three studies are presented in Table 2 and the results of the regression analysis are presented in Table 3.

**Table 2:** **Means and standard deviations of evaluation of initiatives presented with masculine or feminine forms for gender and non-gender related initiatives according to participant gender across all three studies**.

		**Gender initiative**				**Non-gender initiative**			
		**Women**		**Men**		**Women**		**Men**	
		***M***	***SD***	***M***	***SD***	***M***	***SD***	***M***	***SD***
Study 1	Feminine forms	64.33	29.15	40.42	27.70				
	Masculine forms	59.21	26.44	52.19	32.71				
Study 2	Feminine forms	3.73	1.37	2.62	1.25	4.35	1.10	3.87	1.54
	Masculine forms	4.01	1.51	3.13	1.55	4.12	1.07	3.85	1.32
Study 3	Feminine forms	4.62	1.50	4.21	1.68	5.01	0.96	4.43	1.31
	Masculine forms	4.26	1.53	3.67	1.82	4.52	1.40	3.97	1.67

*All means were adjusted for the covariate used in the analysis namely political views*.

**Table 3:** **Study 1. Regression model for the evaluation of the initiative**.

	**ΔR^2^**	**B**	**SE B**
MODEL 1			
Intercept		41.76^***^	3.68
Linguistic Form (LF)		−2.91	3.82
Participant Gender (PG)		14.55^***^	3.86
Support for the parity act		22.79^***^	4.24
MODEL 2	0.02^*^		
Intercept		47.15^***^	4.31
Linguistic Form (LF)		−13.08^*^	5.77
Participant Gender (PG)		5.20	5.53
Support for the parity act		22.99^***^	4.19
LF × PG		17.78^*^	7.62

* *p < 0.05*,

*** *p < 0.001*.

*Model 1: Adjusted R^2^ = 0.17; F_(3, 201)_ = 15.45; p < 0.001*.

*Model 2: Adjusted R^2^ = 0.19; F_(4, 200)_ = 13.20; p < 0.001*.

Study 1 showed that the gender-related social initiative was evaluated less favorably by men when framed in a feminine than in a masculine form. However, no such difference was observed for women. Earlier studies on gender-fair language already observed that men are less supportive of gender-fair language (Jacobson and Insko, 1985; Matheson and Kristiansen, 1987; Parks and Roberton, 2002, 2004), and our results are consistent with these findings. Moreover, it must be emphasized that Study 1 was performed at a time when a heated debate on quotas was ongoing in Poland. Several issues regarding gender equality were raised at the time, and gender was a salient concept. This may have increased the intergroup divides between men and women as well as men's opposition to gender-fair language, which is often mediated by attitudes toward women in general (Parks and Roberton, 2004). However, a serious limitation of Study 1 is that the social initiative presented was about gender equality. This topic may have reinforced the effect of feminine forms in the description. Language reform in the direction of gender-fairness was indeed a political act and originated from the feminist movement (Pauwels, 2003). Thus, novel feminine forms used in a gender context may be perceived as signaling feminism. This could be problematic, since even individuals who support gender equality often avoid calling themselves feminists, as reflected in utterances such as “I'm not a feminist but …” (Buschman and Lenart, 1996; Williams and Wittig, 1997; Burn et al., 2000).

In general, if gender-fair language is perceived as questioning traditional gender arrangements, negative effects should occur mostly in connection with gender issues. However, if gender-fair language is rejected solely because of its novelty, then the effect observed in Study 1 should be independent of the goal of an initiative. In Study 1 the support for a social initiative might have been influenced by both, the linguistic form and the readiness to accept gender quotas. Study 2 was designed to address this possible confound.

## Study 2

Study 2 aimed to replicate the effect of linguistic form found in Study 1. In addition, it examined the question of whether the goal of the initiative, a gender-related vs. other issue, moderated the effect. To avoid associations with in-group interests and to stay clear of ongoing debates about quotas, the gender-related issue in Study 2 involved women professionals helping young female students. The non-gender-related goal was helping students from countries affected by war.

### Methods

#### Participants

Study 2 was again conducted online in Poland and was advertised in the academic forums of two universities in Warsaw. The website of the study was accessed by 744 persons. However, many individuals left the page without completing it; thus, the final sample consisted of 577 students (474 women, 103 men; mean age = 25.50, *SD* = 6.40 years).

#### Measures and procedure

The study was presented as a part of a research project investigating “possibilities for the development of the system of higher education in Poland.” The announcement described the study as a 5-min survey concerning the development of the Polish system of higher education. Participants were to evaluate a grassroots campaign that concerned the system of higher education. To support the cover story, the initiative was described in the layout of a popular opinion magazine in Poland. The initiative supported affirmative action either for women or for students from countries affected by war. The initiative was presented as follows:

“Female leaders, including many lawyers, psychologists, and academics, have proposed the introduction of scholarships and additional positions in the areas favored by the Ministry of Higher Education^4^ for women/students from countries at war. According to psychologist Magda Leska, initiator of the campaign, this would promote the development of economic life, science, and factual gender equality [gender goal] vs. equality [non-gender goal] in access to higher education and the labor market in the world.”

Similar to Study 1, the female proponents of the fictitious initiatives were referred to either with the masculine or the feminine form of their professional title; correspondingly, reference was made to either *psycholog Magda Leska-inicjator akcji* (masculine forms) or to *psycholożka Magda Leska-inicjatorka akcji* (feminine forms).

After reading the introduction, participants were asked to evaluate the proposal by answering seven questions. Participants indicated whether the initiative (1) was generally popular, (2) was governed by genuine concern for other people, (3) was good for the system of higher education; and had the potential of increasing (4) the prestige of higher education in Poland, (5) the quality of schooling, (6) the competitiveness of Polish institutions of higher education, and (7) should be implemented at all Polish institutions of higher education. Answers to these questions could vary from 1 (definitely not) to 7 (definitely yes)^5^. The answers were averaged to form a scale evaluation for the initiative (α = 0.94), which served as dependent measure. In contrast to Study 1, we also measured participants' political attitudes (one item with answers from 1 (very liberal) to 7 (very conservative). Moreover, we asked their opinions on factors influencing women's positions in the job market. For this purpose, we provided seven items from the Neosexism Scale (Tougas et al., 1995), which included such items as “Women will make more progress by being patient and not pushing too hard for change.” After recoding several items, we combined the items into a reliable scale (α = 0.74) that captured participants' political attitudes, including gender-related features. Finally, participants were asked for their comments and were provided with debriefing information about the study.

### Results and discussion

Similar to Study 1, we conducted a regression analysis. In the first step, we used linguistic form (coded 0 for masculine and 1 for feminine), goal of the initiative (0 for non-gender and 1 for gender), and participant gender (0 for male and 1 for female) as predictors. As in Study 1, we included participants' political views (mean-centered) as a covariate in the analysis. In the second step, we added three two-way interaction terms derived by multiplying the initial predictors; and in the third step, we added one three-way interaction term. The analysis of the full model with all two-way interactions and the three-way interaction of participant gender, linguistic form, and goal of the initiative revealed that the three-way interaction did not improve the model, ΔR^2^ = 0.000. Results of the regression analysis are presented in Table 4. Because we predicted only the two-way interaction of linguistic form and goal of the initiative and that the interaction of participant gender and linguistic form observed in Study 1 would be replicated, we used Model 2 in the Hayes (2012) SPSS macro, testing the interactions of the focal predictor (linguistic form) with the two remaining factors^6^. The hypothesized interaction of linguistic form and goal of the initiative was significant and indicated that the conditional effect of linguistic forms was close to significant for the gender initiative: *b* = −0.27, *SE* = 0.15; *p* = 0.07. The gender initiative was evaluated less favorably when presented with feminine forms than when masculine forms were used. However, evaluation of the non-gender initiative was not affected by linguistic form: *b* = 0.21, *SE* = 0.15; *p* = 0.16.

**Table 4:** **Study 2. Regression model for the evaluation of the initiative**.

	**ΔR^2^**	**B**	**SE B**
MODEL 1			
Intercept		3.82^***^	0.15
Linguistic Form (LF)		−0.03	0.11
Participant Gender (PG)		0.43^**^	0.14
Goal of the Initiative (GI)		−0.46^***^	0.11
Political views		−0.36^***^	0.06
MODEL 2	0.02^**^		
Intercept		4.03^***^	0.22
Linguistic Form (LF)		0.07	0.27
Participant Gender (PG)		0.03	0.24
Goal of the Initiative (GI)		−0.81^**^	0.28
Political views		−0.36^***^	0.06
LF × PG		0.16	0.28
LF × GI		−0.47^*^	0.21
PG × GI		0.71^*^	0.28

* *p < 0.05*,

** *p < 0.01*,

*** *p < 0.001*.

*Model 1: Adjusted R^2^ = 0.11; F_(4, 570)_ = 19.28; p < 0.001*.

*Model 2: Adjusted R^2^ = 0.13; F_(7, 567)_ = 12.93; p < 0.001*.

For the gender initiative, Study 2 replicated the results of Study 1 and showed that both male and female participants evaluated the initiatives less favorably when it was framed in the feminine than in the masculine form. Having documented the negative effects of gender-fair language in a country where this linguistic usage is novel, we then examined the effects in a country where use of gender-fair language is already well established.

## Study 3

Study 3 was conducted in Austria to clarify whether the long-term practice of gender-fair language is reflected in positive reactions to this usage in the evaluation of social initiatives.

### Methods

#### Participants

Study 3 was conducted online and was advertised in Austrian forums and via email. We offered participants the opportunity to take part in a lottery for five 10-Euro vouchers. The website of the study was accessed by 309 individuals; the final sample of those who completed the study comprised 210 students (113 women, 96 men, and one individual who did not provide information on gender). To ensure that the participants had sufficient linguistic competence to notice the subtle linguistic manipulation, we excluded four individuals whose native language was not German from further analysis. Thus, the final sample consisted of 206 students (110 women, 95 men, *M age* = 31.92, *SD* = 9.48 years).

#### Measures and procedure

The announcement described the study as a 5-min survey on the development of the system of higher education in Austria. In daily life, it is quite common to use academic titles when introducing people. In Austria, the feminization of academic titles is available (Universitätsgesetz, 2002), although masculine forms were used for women in former times. Therefore, the woman in the social initiative was either introduced as “Dr. Martina Winkler (Psychologe)” (masculine forms) or as “Dr. Martina Winkler (Psychologin)” (feminine forms) “Dr. Martina Winkler (psychologist).”

The initiative was evaluated using five questions, a shortened version of the scale used in Study 2^7^. Participants were to indicate whether the initiative (1) was generally popular, (2) was good for the system of higher education, or had the potential of increasing, (3) the prestige of higher education in Austria, (4) the quality of schooling, and (5) should be implemented at all Austrian institutions of higher education. Answers to these questions could vary from 1 (definitely not) to 7 (definitely yes). The answers were averaged to form a scale evaluation of the initiative (α = 0.90), which served as dependent measure. Participants also answered the yes/no question “Would you support this initiative if it were to be implemented at your university?” Similar to Study 2, we assessed participants' political attitudes [one item with answers from 1 (very liberal) to 7 (very conservative)]. Moreover, we asked their opinions on factors influencing the situation of women. Thus, we provided seven items from the gender-specific system justification scale (Jost and Kay, 2005, adapted for German by Ullrich and Cohrs, 2007)^8^. After recoding several items, we combined them into a reliable scale (α = 0.78; one item was removed due to very low inter-item correlations), which captured participants' political attitudes, including their attitudes on gender-related issues. Finally, participants were asked for demographical data and for their comments and were provided with information about the study^9^.

### Results and discussion

We conducted a regression analysis. In the first step, we used linguistic form (coded 0 for masculine and 1 for feminine), goal of the initiative (0 for non-gender and 1 for gender), and participant gender (0 for male and 1 for female) as predictors. Similar to Studies 1 and 2, we included participants' political views (mean-centered) as covariates in the analysis. In the second step, we added three two-way interaction terms derived by multiplying the initial predictors, and in the third step, we added one three-way interaction term. The analysis of the full model with all two-way interactions and the three-way interaction of participant gender, linguistic form, and goal of the initiative revealed that neither the second (Δ*R*^2^ = 0.001) nor the third iterations (Δ*R*^2^ = 0.000) improved the model. Results of the regression analysis are presented in Table 5. The results in the first iteration showed that an initiative presented in gender-fair language was evaluated more positively than an initiative presented in the masculine.

**Table 5:** **Study 3. Regression model for the evaluation of the initiative**.

	**B**	**SE B**
MODEL 1		
Intercept	4.12^***^	0.22
Linguistic Form (LF)	0.47^*^	0.20
Participant Gender (PG)	0.34	0.21
Goal of the Initiative (GI)	−0.35^*^	0.20
Political views	−0.33^***^	0.10

* *p < 0.05*,

*** *p < 0.001*.

*Model 1: Adjusted R^2^ = 0.10; F_(4, 200)_ = 6.42; p < 0.001*.

Study 3 was conducted in Austria, a country where, in contrast to Poland, gender-fair language is well established in everyday life. The results demonstrate that in German, gender-fair language has lost its association with feminism because there were practically no differences in favorability due to the linguistic forms used. There was a gender difference in the evaluation of the initiatives, as men rated the initiatives less favorably than women. However, more important was the finding that initiatives received better evaluations when feminine forms were used for the female proponents than masculine forms regardless of participant gender. This indicates that the use of masculine forms in referring to women appears odd when speakers are accustomed to gender-fair language, even if masculine generics were formerly common in the respective country.

## General discussion

The present research used an indirect approach to examine socially motivated linguistic change and more specifically, changes in the use of gender-related forms. For this purpose, we compared the effects of gender-fair language in Poland and Austria. Although grammatical gender languages are spoken in both countries, they differ considerably in the use of gender-fair language. While this usage is well established in Austria, it is relatively novel in Poland. Across the first two studies, our results show that in a country where gender-fair language is not common (Poland), social initiatives are evaluated less favorably when gender-fair (i.e., feminine) vs. traditional masculine forms are used. In Study 1, Polish men (but not women) evaluated the initiative for gender quotas on election lists less favorably. Study 2 replicated this effect for both male and female participants. In addition, it showed that the effect depended on the goal of the initiative. Feminine job titles led to less favorable evaluations of the initiative when its goal was gender equality (support of female students), but not when the initiative was aimed at achieving other forms of equality (supporting students of countries affected by war).

Consistent with other studies on German (Vervecken and Hannover, 2012), Study 3 on Austrian German showed that designating women with gender-fair (feminine) forms led to higher support for all types of initiative than when the female proponents were labeled with masculine forms. In line with Vervecken and Hannover (2012), we assumed that the use of gender-fair language in German is currently associated with higher education or competence and has lost its novelty as well as its associations with feminism. Violating a linguistic norm, as well as the gender-fairness norm, may be considered a sign of incompetence (e.g., Giles and Coupland, 1991) and is thus stigmatized. Moreover, the positive effect of gender-fair language, especially of feminine forms referring to a group of women only, on evaluations of the initiative suggests that this usage has become so familiar to speakers of (Austrian) German that failing to use it decreased participants' support for the initiative. Although we do not have direct evidence, participants made several comments in that direction. For instance, some of those who read the text with the masculine forms commented that “the wording was wrong, because masculine forms were used although this was about women!” or “It is very irritating for me that you used masculine forms for women!”

Our studies are the first to investigate different stages in the implementation of gender-fair language by applying the same research design in two different countries. Although we did not directly study the effects of language policies or familiarity with gender-fair language, our results helped elucidate the changes in reactions to gender-fair language and approximate the process that occurs over time as a language changes. When gender-fair language is new, it may face general resistance as it is unfamiliar to speakers and may be perceived as hampering the fluency of everyday speech. Past research suggests that objections to gender-fair language are predominantly due to its novelty (Blaubergs, 1980; Parks and Roberton, 1998). In addition, this usage can be associated with feminism since feminists have fiercely advocated its use in public discourse (see Blaubergs, 1980; Parks and Roberton, 1998). Proponents of gender-fair language were also judged as overly sensitive and preoccupied with non-essential matters (Parks and Roberton, 1998). This was corroborated by the observation that proponents of gender-fair language have been subject to “hostility and ridicule.” Participants in the Parks and Roberton (1998) study, for example, believed that …” the only people who really take offense to any such things are the feminist activists who do nothing but protest all day long” (p. 453). Arguments of this kind were uttered not only by students (Parks and Roberton, 1998) but also in the scientific community (see Maass et al., 2014). Thus, we assume that in the period following the introduction of gender-fair language, its co-occurrence with a gender equality issue may be perceived as strongly indicating a feminist position. Opposition to gender-fair language may be particularly strong in a gender equality context, where both the topic of discussion and language use may suggest a feminist stance. However, when either gender-fair language or a gender-equality issue is presented separately, the association with feminism may be sufficiently unobtrusive to not affect evaluations of the social cause. In an advanced stage, when the use of gender-fair forms has become standard, gender-fair wording is likely to be evaluated as positively as traditional language—or even more positively, once the habit of referring to a woman in the masculine becomes outdated (cf. “policeman Anne Schmidt”).

Our findings may offer an explanatory framework for the results of earlier studies, which report both positive and negative speaker perceptions of gender-fair wording (e.g., McConnell and Fazio, 1996; Vervecken and Hannover, 2012). These seemingly contradictory results reflect different stages of adaptation to gender-fair language in the respective societies investigated. In the 1980s, when gender-fair language was new everywhere, the negative effects of gender-fair forms probably occurred regardless of the topic of under discussion as a spill-over effect. However, in our studies, we did not find such negative effects on the non-gender context in Poland, which may suggest that this country is already on its way to adopting, or at least accepting, gender-fair language. Using gender-fair language outside the feminist context may help to make it “normal.” Reformed language may then contribute to gender equality. The negative effect of gender-fair language for gender-related initiatives described for Poland can be considered temporary and can be assumed to persist until gender-fair language has become more common and less associated with the feminist context. This development appears to have occurred in Austrian German. Nevertheless, future studies should try to capture the change in attitudes toward gender-fair language more directly. Additionally, future studies should tackle other samples and languages in order to assess the generalizability of the obtained effects.

The most important conclusion to draw from our studies is that language policies aiming at political correctness should not be evaluated rashly. As Bob Dylan said, the times they are a-changin'. Accordingly, negative attitudes toward reformed language may become more positive. What once was new may then become the norm. This conclusion may be helpful for activists and policymakers when advocating changes that at first appear to have detrimental side-effects.

### Conflict of interest statement

The authors declare that the research was conducted in the absence of any commercial or financial relationships that could be construed as a potential conflict of interest.

## Footnotes

^1^http://gendering.codeplex.com/

^2^To assess behavioral intentions, we also asked participants whether they would support the presented social initiative with their signature, with possible answers being no (coded as 1), I don't know (coded as 2), and yes (coded as 3). When this variable was used as a dependent variable, the pattern of results matched those presented in the main analysis. The interaction term was significant at the 0.09 level.

^3^To examine the independence of the covariate and other predictors, a logistic regression was used to test whether the log odds of support for the parity act depended on experimental condition and participant gender as well as their product term. The overall regression was not significant, $\chi_{\left(3\right)}^{2}=1.26$, *p* = 0.74, Nagelkerke *R*^2^ = 0.01. This indicates that support for the parity act was similar across genders and experimental conditions and the use of covariate was justified. Information on the support for parity act was provided by 205 participants.

^4^Most often, majors in technical and natural sciences receive financial support from the Ministry of Higher Education to ensure there are enough suitably qualified experts in strategically important economic domains.

^5^To assess behavioral intentions, we asked participants whether they would support the presented social initiative with their signature, possible answers being no (coded as 0) and yes (coded as 1).

^6^The remaining two-way interaction of goal of the initiative and participant gender was also significant. The non-gender initiative was evaluated similarly by both genders *b* = 0.10, *SE* = 0.19; *p* = 0.62, whereas the gender initiative was evaluated more favorably by female than male participants *b* = 0.83, *SE* = 0.21; *p* < 0.001.

^7^In comparison to Study 2, two questions were omitted. One question (assessing the competitiveness of Polish institutions of higher education) was omitted because we have encountered considerable problems with translation and back-translation of this item into German. Second question (whether the initiative was governed by genuine care for other people) was omitted because we have included two additional similar questions examining for exploratory purposes judgments of fairness of the initiative (see also Footnote 9).

^8^In Study 2 we have used a scale that was previously used in Poland (Formanowicz et al., 2013). However, for the German language we referred to the related scale for which the translation was available for us.

^9^For exploratory purposes, we also examined judgments of fairness (“Is this initiative fair?” and “Does it contribute to the public good?”; α = 0.78), of the annoyance the initiative might cause (“Could anybody be annoyed by this initiative?” and “Is this initiative irritating?”; α = 0.78), and associations of feminism. In addition, we applied a scale measuring attitudes toward affirmative action programs (Bell et al., 2000; α = 0.84). Responses to the latter were in the format of a semantic differential and could vary, for instance, from 1 (negative) to 5 (positive). Finally, participants were asked how strongly they supported feminism.
