# Supplementary material for: Capturing socially motivated linguistic change: how the use of gender-fair language affects support for social initiatives in Austria and Poland
Source: Front Psychol. 2015 Oct 31;6:1617. doi: 10.3389/fpsyg.2015.01617 (PMC4628104; doi:10.3389/fpsyg.2015.01617)
Supplement: Supplementary file 1 [file Original_manipulations.PDF]

Appendix A. Original manipulations used across all three studies. All the manipulated texts are bold and presented in the feminine form.

Study 1

Zbliżają się Wybory Samorządowe i niebawem upłynie termin składania zawiadomień o utworzeniu komitetu wyborczego i zamiarze zgłaszania kandydatów na radnych.

Ustawa o parytetach na listach wyborczych (według której 50% miejsc na listach wyborczych przysługiwałoby kobietom) nie została jeszcze rozpatrzona przez Sejm.

Środowisko kobiece **(w tym wiele znanych adwokatek, psycholożek, socjolożek i nauczycielek akademickich)** rozważa wystosowanie apelu o wspieranie oddolnej inicjatywy wpisywania na listy wyborcze większej liczby kobiet, zgodnie z Ustawą o parytecie.

Według projektu apelu, umieszczenie przez dane ugrupowanie na liście wyborczej 50% kobiet byłoby sygnałem realnego poparcia dla idei równego statusu kobiet i mężczyzn w nowoczesnej Polsce.

Study 2 – gender related goal

Środowisko kobiece **w tym wiele znanych adwokatek, psycholożek i nauczycielek akademickich** zaproponowało by wprowadzić dla kobiet stypendia i dodatkowe miejsca na tzw. „kierunkach zamawianych". Miałoby to zdaniem **psycholożki** Magdy Leskiej - **inicjatorki** akcji, promować udział kobiet w życiu ekonomicznym i naukowym oraz sprzyjać faktycznemu równouprawnieniu płci w szkolnictwie wyższym i na rynku pracy.

Study 3 – gender related goal

Frauen in führenden Positionen, darunter viele **Soziologinnen, Anwältinnen und Professorinnen** der österreichischen Universitäten haben vorgeschlagen zusätzliche Stipendien für Frauen in den Bereichen IT und Technik einzuführen. Gemäß **Dr.$^{\mathbf{in}}$** Martina Winkler (**Psychologin**), die diese Initiative ins Leben gerufen hat, würde dies nicht nur die aktive Beteiligung von Frauen in Wirtschaft und Wissenschaft fördern, sondern es wäre eine Maßnahme zur tatsächlichen Gleichstellung für den Zugang zu höherer Bildung und am Arbeitsmarkt.
